# Robust, conformal Cu_2_O coatings on polypropylene fabrics *via* atmospheric-pressure spatial atomic layer deposition

**DOI:** 10.1039/d6na00121a

**Published:** 2026-05-01

**Authors:** Guvanch Gurbandurdyyev, Sarah Khalid, Samantha Lum, Fan Ye, Autumn Cheon, Kam Chiu Tam, Stephanie DeWitte-Orr, Kevin P. Musselman

**Affiliations:** a Department of Mechanical and Mechatronics Engineering, University of Waterloo Waterloo ON Canada; b Waterloo Institute for Nanotechnology Waterloo ON Canada kevin.musselman@uwaterloo.ca; c Department of Health Sciences, Wilfrid Laurier University Waterloo Ontario Canada; d Department of Chemical Engineering, University of Waterloo Waterloo ON Canada

## Abstract

Copper-based nanocoatings offer significant potential for advanced functional textiles due to their broad-spectrum antimicrobial properties. For the first time, this study demonstrates atmospheric-pressure spatial atomic layer deposition (AP-SALD) as a scalable method for depositing uniform cuprous oxide (Cu_2_O) coatings on a porous textile substrate. The AP-SALD process enables conformal, nanoscale coating of spun-bond polypropylene fabrics – commonly used in N95 respirators – at polymer-compatible temperatures (100–120 °C), eliminating the need for vacuum systems. Structural and morphological characterization confirms the formation of continuous Cu_2_O films with growth rates of ∼0.06 nm per cycle, confirmed *via in situ* optical monitoring. The coatings exhibit excellent mechanical durability under abrasion, washing, and flexing, with no significant damage or delamination. The fabric's filtration performance remains unaffected after coating and coatings deposited at 100 °C and 120 °C demonstrate low cytotoxicity in biocompatibility assays, supporting their suitability for skin-contact applications. Together, these results establish AP-SALD as a practical, industrially scalable, and high-throughput route for producing durable and safe functional textiles, with direct relevance to healthcare and personal protective equipment.

## Abstract 2

Atmospheric-pressure spatial atomic layer deposition produces conformal, robust Cu_2_O coatings on polypropylene textiles at polymer-compatible temperatures. The nanoscale coatings show durability and biocompatibility, without affecting breathability.

## Graphical abstract

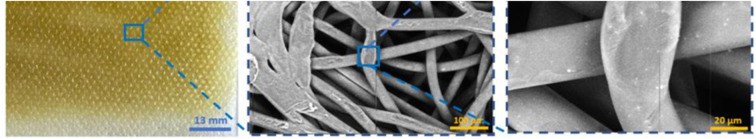

## 1. Introduction

Functionalizing textiles with nanomaterials has become increasingly important due to growing demand for advanced fabrics in healthcare, electronics, protective equipment, and consumer apparel.^1,2^ Among various functional nanomaterials, copper-oxide-based coatings are attractive because of their unique combination of properties: strong UV-blocking and photocatalytic activity, semiconducting behavior, excellent biocompatibility and non-toxicity.^3–5^ Moreover, copper and its oxides are well-known powerful broad-spectrum antimicrobials (effective against bacteria and viruses). Cuprous oxide (Cu_2_O), in particular, has been found to have superior long-term durability and more consistent antimicrobial performance, compared to elemental copper and cupric oxide (CuO).^5–7^ These characteristics make Cu_2_O an appealing coating for fabrics intended for applications such as antimicrobial hospital textiles or protective face masks.

Various methods have been explored to deposit coatings, including Cu_2_O, on textiles. Each has trade-offs in coating quality, durability, and scalability, as discussed in past review articles.^8–12^ Wet-chemical techniques such as dip-coating, sol–gel processing, and hydrothermal growth are widely used due to their simplicity and low cost; however, they often suffer from poor uniformity and weak adhesion, particularly on hydrophobic polymer fibers like polypropylene.^13–15^ Vacuum-based approaches like atomic layer deposition (ALD) and magnetron sputtering offer exceptional control over film thickness and conformity, and have been successfully applied to coat fibrous substrates with nanoscale oxides.^16,17^ Yet, such methods are constrained by high equipment costs, slow deposition rates, and the need for vacuum and elevated temperatures, making them impractical for high-throughput or thermally sensitive textile applications. The adhesion of sputtered coatings also depends heavily on the deposition parameters and properties of the textile surface.^11^*In situ* approaches, where Cu_2_O nanoparticles are embedded directly into polymer masterbatches during fiber extrusion, have demonstrated excellent adhesion and wash durability, but these require upstream modification of fiber manufacturing, which can be costly, and are not compatible with existing fabrics.^18^ Across coating strategies, ensuring strong adhesion is critical to prevent copper leaching, which can not only reduce antimicrobial efficacy but also raise safety concerns.^19^

In this work, these challenges are addressed by utilizing atmospheric-pressure spatial atomic layer deposition (AP-SALD) to deposit Cu_2_O coatings directly on spun-bond polypropylene (PP) fabric. AP-SALD is an emerging technique that decouples the ALD half-reactions in space rather than time, enabling continuous, roll-compatible deposition at atmospheric pressure.^20^ By eliminating vacuum chambers and replacing purge steps with a constant-flow reactor design, AP-SALD can achieve orders-of-magnitude higher growth rates than temporal ALD.^21^ It has previously been applied to rapidly grow metal oxide films on rigid substrates for electronics and energy devices,^22^ but its application to coating flexible, porous, textile materials with copper oxides has not been widely studied.^23^ This study demonstrates that AP-SALD can produce conformal Cu_2_O nanoscale coatings wrapping each fiber of a nonwoven fabric, effectively forming a “shell” of Cu_2_O around the polymer fibers. By careful selection of deposition parameters (precursor delivery, substrate temperature, stage speed, and number of deposition cycles), the desired Cu_2_O phase is achieved at low temperatures compatible with the PP fabric. The resulting coatings exhibit excellent adhesion and mechanical robustness. It is also shown that Cu_2_O-coated fabric retains the filtration performance (air permeability) of the material – a critical requirement for applications in N95 or other respirators – and shows no cytotoxicity in biocompatibility assays.

Overall, the outcomes of this research establish AP-SALD as a high-throughput, industrially scalable method for producing Cu_2_O-coated fabrics with a level of uniformity and durability previously achievable only with laborious vacuum processes. By bridging the gap between laboratory nanocoatings and real-world textile performance requirements, this work opens new possibilities for multifunctional textiles in healthcare and personal protective equipment.

## 2. Results and discussion

### 2.1. AP-SALD of Cu_2_O on polypropylene fabrics

**Fig. 1:**
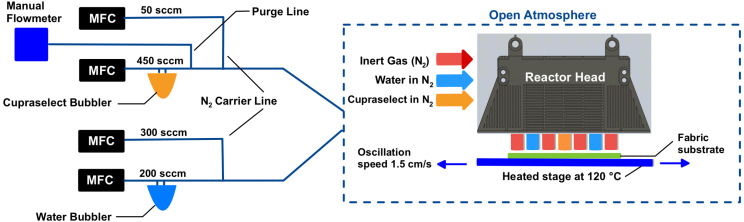
Schematic of the AP-SALD method, including a schematic of the reactor head, which shows the continuous flow of the precursors: Cupraselect (orange) and water (blue). Flows were controlled using mass flow controllers (MFC). The precursors were bubbled with high-purity nitrogen and combined with nitrogen carrier-gas flows to deliver them to the reactor head.

Cu_2_O films were deposited on spun-bond polypropylene fabric (the outer-layer material of N95 respirators) using a custom AP-SALD system. Fig. 1 shows a schematic of the AP-SALD reactor head with spatially separated precursor flows: Cupraselect (orange) and H_2_O (blue) are injected in alternating zones, while nitrogen curtains (red) confine them to prevent gas-phase intermixing. The fabric substrate was taped onto a heated stage and oscillated back-and-forth under the head. Each back-and-forth oscillation of the fabric can deposit up to two monolayers of the Cu_2_O film as the substrate sequentially encounters the Cupraselect and H_2_O zones (equivalent to two conventional ALD cycles). By repeating up to a few hundred oscillations, uniform copper-oxide nanocoatings were built up on the fabric.

**Fig. 2:**
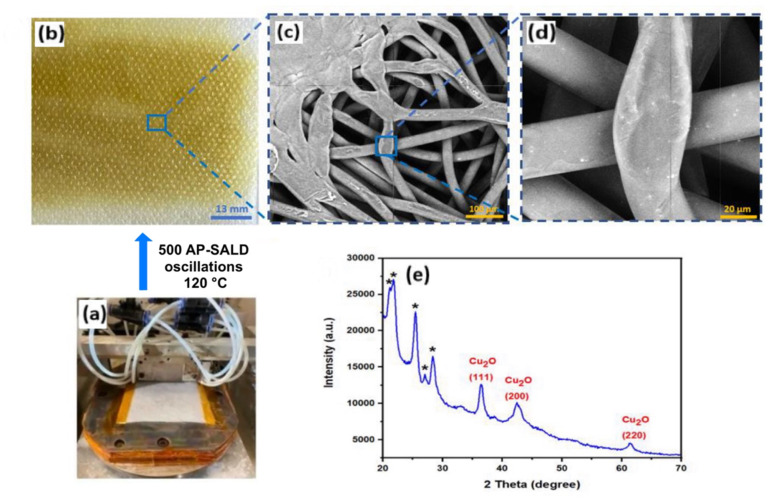
Cu_2_O-coated spun-bond outer polypropylene fabric of N95 respirators (50 g m^−2^). (a) PP fabric on heated stage of the AP-SALD system. (b) PP fabric after Cu_2_O coating with 500 AP-SALD oscillations (1000 AP-SALD cycles) at 120 °C. (c) Low-magnification and (d) high-magnification SEM images of coated PP fabric. The conductive Cu_2_O coating enables imaging of the insulating PP fibers. (e) XRD of coated PP fabric showing the formation of Cu_2_O (peaks labelled with “*” correspond to the PP).

To ensure even coating on the porous textile, it was found effective to stack two layers of fabric during deposition, with the target sample as the bottom layer. This is shown in Fig. S1 (see SI). The two-fabric configuration minimized direct precursor blow-through and ensured that the bottom layer received a full exposure to reactants. All subsequent characterizations were performed on the bottom fabric from such two-layer depositions. The fabric was maintained under slight tension on a glass backing plate to keep it flat; insufficient tension can lead to tenting or billowing of the fabric, allowing precursors to bypass parts of the substrate and causing non-uniform deposition. After 500 AP-SALD oscillations (1000 AP-SALD cycles) at 120 °C, the PP fabric changed from white to a pale yellowish-brown color, indicating the presence of Cu_2_O on the fibers (shown in Fig. 2a and b). The coloration was homogeneous across the fabric with no obvious crusts or uneven patches.

Scanning electron microscopy (SEM) imaging confirmed that the Cu_2_O coating enveloped the fibers with a conformal nanoscale layer. Fig. 2c and d present SEM micrographs of a Cu_2_O-coated PP fabric at low and high magnifications, respectively. Notably, no isolated particles or aggregates are observed on the fibers at the accessible magnifications, consistent with the smooth coatings expected for AP-SALD. Uncoated fabrics could not be imaged due to charging of the insulating polypropylene. The clear imaging of the fibers in Fig. 2c and d confirms the presence of conductive Cu_2_O along the entire length of all fibers. The Cu_2_O was visible on both sides of the fabric, suggesting that the coating forms a continuous shell around the fiber circumference, as expected for AP-SALD.^24^ However, it is noted that discontinuities in the coatings may be present that are not visible by SEM, *e.g.*, at touchpoints between adjacent fibers where the AP-SALD precursors may not be effectively delivered to the fiber surfaces. Given the non-polar nature of the polypropylene fiber surfaces, which have few active sites to initiate growth, the coating nucleation is expected to occur *via* subsurface infiltration, as reported previously for polypropylene.^25,26^ The chemical precursors diffuse into the near-surface region of the propylene fibers, where they react and become trapped, as illustrated in Fig. S2. This provides the required nucleation sites for further reactions and the formation of a continuous coating. The fibrous morphology of the substrate may also aid in trapping precursor vapors, facilitating nucleation around the fibers.

Cross-sectional SEM imaging (Fig. S3) was conducted to further characterize the Cu_2_O coatings. Initially, fabric samples were immersed in liquid nitrogen to increase brittleness and facilitate clean cross-sectional fractures. However, this approach proved ineffective, as fibers underwent plastic deformation rather than fracturing cleanly as shown in Fig. S3a. An alternative method was then employed: encapsulating coated fabrics in epoxy resin to enhance rigidity and enable clean cross-sectioning. Since the epoxy-embedded fabrics were not brittle enough to be cleanly fractured, they were cut into pieces and analyzed with SEM. These SEM images (Fig. S3b) indicate some change in contrast at the fiber periphery, consistent with the continuous Cu_2_O coatings observed above. In summary, AP-SALD produces a conformal Cu_2_O nanocoating on the PP textile, covering all visible fiber surfaces uniformly, even the curved and interwoven fiber surfaces.

### 2.2. Crystal structure and phase analysis

Understanding the phase and crystallinity of the deposited copper oxide is crucial, as Cu^+^ and Cu^2+^ oxides have different properties.^27^ X-ray diffraction (XRD) analysis was used to determine the phase of AP-SALD coatings on PP fabric. As shown in Fig. 2e, the diffraction peaks observed at 2*θ* ≈ 36.5°, 42.3°, and 61.4° correspond to the (111), (200), and (220) planes of Cu_2_O (JCPDS No. 05-0667), confirming a cubic cuprite phase. Peaks near 16.6° arise from the semi-crystalline PP substrate. Crucially, no diffraction peaks associated with CuO were detected, indicating that the deposited film is primarily Cu_2_O. Within the detection limits of the XRD, no crystalline CuO or metallic Cu phases were observed on the coated fabric samples.

Deposition temperature is a key factor governing the Cu_2_O *vs.* CuO composition. Literature on AP-SALD and related chemical vapor deposition (CVD) methods indicates that lower substrate temperatures favor Cu_2_O, whereas higher temperatures (typically >200 °C) can yield CuO or even Cu metal due to precursor decomposition.^28,29^ This trend was confirmed experimentally: coatings formed at 100 °C and 120 °C showed only Cu_2_O peaks, while films deposited at 200 °C on glass substrates displayed a new broad peak around 50.5°, potentially indicating Cu or a non-cuprous impurity phase (see Fig. S4a and b). However, since 200 °C is above the melting point of PP (165 °C), fabric coatings were only pursued up to 120 °C in this study.

To complement the XRD phase identification, X-ray photoelectron spectroscopy (XPS) was performed on Cu_2_O films deposited on glass (see Fig. S4e and f). The XPS Cu 2p spectra show the characteristic Cu^+^ features: Cu 2p_3_/_2_ at ∼932.5 eV with a very small satellite, and Cu 2p_1_/_2_ at ∼952.3 eV. The weak shake-up satellite peaks indicate only a minor presence of Cu^2+^ (CuO), consistent with a mostly Cu_2_O composition. The O 1s spectrum similarly has a dominant peak at ∼530.4 eV (Cu_2_O lattice oxygen) with a smaller shoulder at ∼531.4 eV (which could correspond to O in CuO or surface –OH). These surface-sensitive XPS results suggest that any CuO is limited to perhaps a thin oxidized layer on the outermost surface after air exposure, while the bulk of the film is predominantly Cu_2_O. This is reasonable since the surface of Cu_2_O is known to slowly oxidize to CuO upon long exposure to air and moisture.^29^ In practical use, especially for face masks which are exposed to exhaled moisture, some gradual Cu_2_O to CuO conversion might occur over time. Notably, CuO is also antiviral, though less so than Cu_2_O.^5^

In summary, phase analysis confirms successful low-temperature deposition of Cu_2_O coatings *via* AP-SALD without significant CuO formation. Maintaining the Cu_2_O phase at polymer-compatible temperatures (100–120 °C) is expected to be beneficial for providing the desired antiviral functionality and semiconducting properties (optical bandgap ∼2.5 eV, see UV-Vis data in Fig. S4c and d). This phase purity underscores the suitability of the AP-SALD process for producing functional, low-temperature oxide coatings on polymer substrates.

### 2.3 *In Situ* reflectance and VI model for thickness monitoring

Direct thickness measurement of nanoscale coatings on fibrous, irregular substrates is challenging. Conventional techniques like ellipsometry and profilometry are not easily applied to fabrics,^30^ and SEM cross-sections are difficult to prepare on soft fibers, as noted earlier (Section 2.1). To address this, an *in situ* optical monitoring method during AP-SALD was implemented to estimate the growth per cycle (GPC) in real time. This technique, previously used to monitor ZnO coatings on fabrics,^31^ employs a normal-incidence reflectance probe focused on the substrate during deposition. The growing film causes periodic oscillations in reflectance due to optical interference between reflections from the film surface and film–substrate interface.

These oscillations were analyzed using the Virtual Interface (VI) model developed by Breiland and Killeen,^32^ which fits the reflectance signal *R*(*t*) as a decaying sinusoid. The frequency of oscillations relates to GPC, while the decay reflects absorption or scattering losses. Importantly, the model does not require prior knowledge of the film's refractive index. The method was calibrated on glass substrates first, where independent ellipsometry confirmed the results, then applied to fabric coatings.

**Fig. 3:**
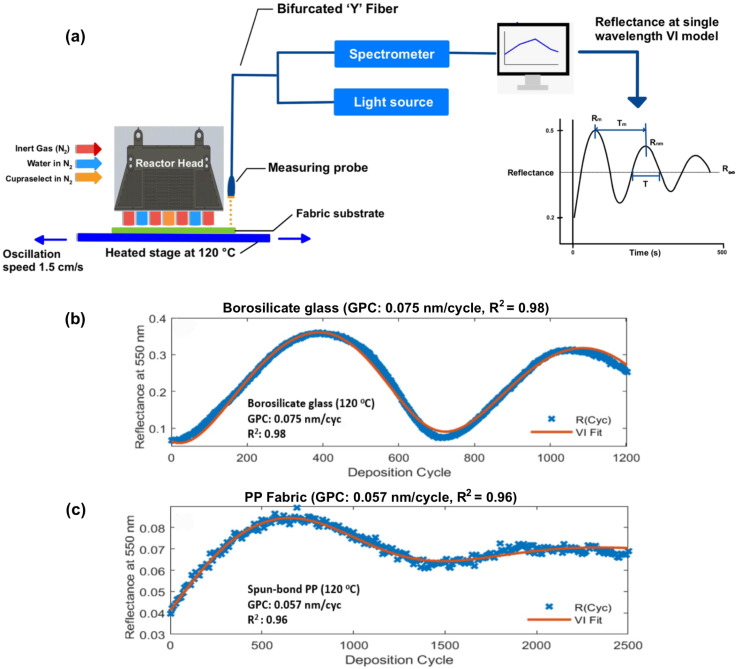
(a) Schematic of the *in situ* reflectometry setup and the theoretical oscillatory plot for the VI method. Periodic oscillations in the reflectance at *λ* = 550 nm (as measured by a reflectance probe and spectrometer) were fit to the VI model to determine the growth per cycle. Measured reflectance intensity at *λ* = 550 nm as a function of deposition cycles for Cu_2_O films coated on (b) borosilicate glass and (c) spun-bond PP fabric at 120 °C. The fit to the VI model is shown in red and the goodness of fit is indicated (*R*^2^). The fits indicated GPCs of 0.075 nm per cycle and 0.057 nm per cycle on glass and PP fabric, respectively.

Fig. 3a shows a schematic of the reflectometry setup and a representative interference curve from the VI model. Fig. 3b and c present the measured reflectance at 550 nm *versus* AP-SALD cycles for Cu_2_O films on glass and PP fabric at 120 °C (top fabric of the two-layer depositions). Both show oscillatory behavior, although the fabric signal (Fig. 3c) exhibits lower contrast and higher noise due to light scattering. Still, clear damped oscillations were observed. Model fits yielded GPC values of ∼0.075 nm per cycle on glass and ∼0.057 nm per cycle on fabric.

These low GPC values reflect the self-limiting nature of ALD. Notably, the rate on PP fabric (0.057 nm per cycle) is only slightly below that on glass (0.075 nm per cycle), despite polypropylene's hydrophobicity and lack of surface –OH groups that typically facilitate ALD nucleation. This may be due to the fabric's porous, high-surface-area structure, enabling precursor access to reactive sites (*e.g.*, at fiber junctions or oxidized regions). Additionally, the porous structure may trap precursors within the fabric, aiding the nucleation and growth. Previous reports for Cu_2_O ALD using similar precursors show lower GPC values (∼0.02–0.03 nm per cycle),^29^ but the higher rates in this study likely result from partial chemical vapor deposition in the AP-SALD reactor. Factors such as precursor mixing in the gas phase at the zone boundaries or increased head–substrate spacing (to accommodate fabric roughness) may enhance growth beyond ideal ALD limits. Despite the higher GPC, the films remain thin; for example, 0.057 nm per cycle over 500 AP-SALD oscillations (1000 AP-SALD cycles) corresponds to ∼60 nm thickness. It is important to note that this reflectance approach measures the coating thickness on the uppermost fabric layer, but some variation in thickness was observed for the top and bottom fabric layers in Fig. S1b. Nonetheless, *in situ* monitoring proved valuable for verifying steady coating growth and estimating film thickness in real time.

### 2.4 Quality assessment of Cu_2_O-coated fabrics

#### 2.4.1. Mechanical robustness and adhesion of coatings

**Fig. 4:**
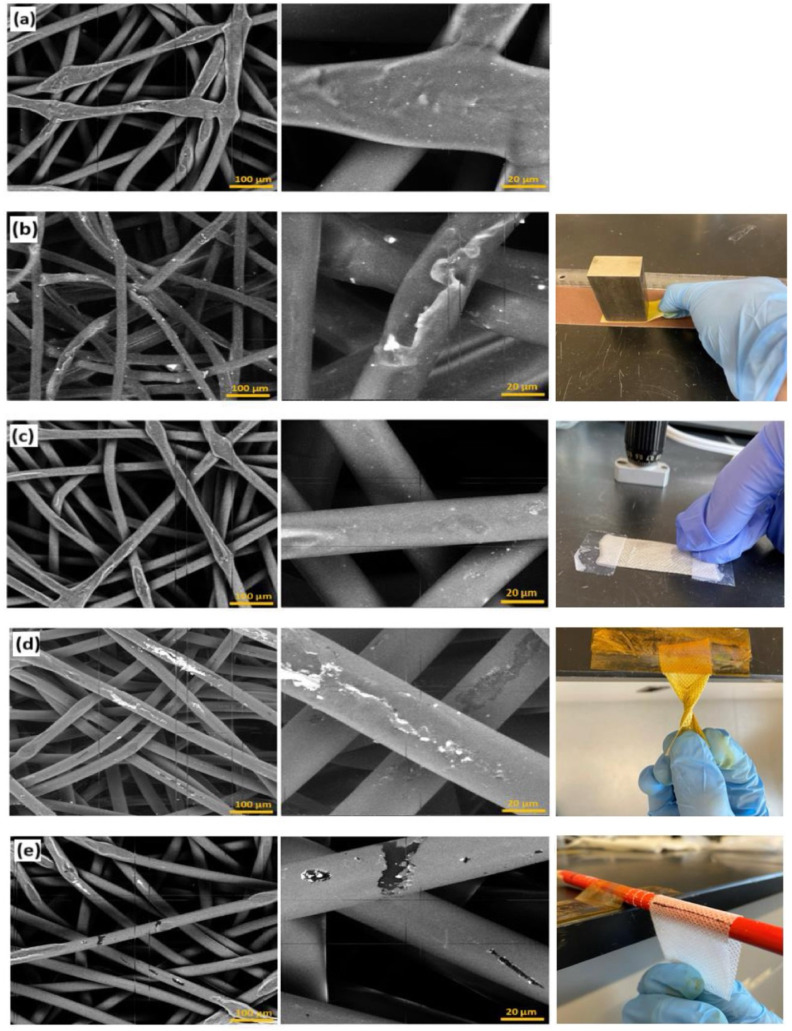
SEM images of Cu_2_O-coated PP fabric after (a) washing, (b) linear abrasion, (c) tape adhesion, (d) twisting and (e) bending tests. Test geometries are shown on the right. The coatings remained largely intact across all tests, with some damage on the uppermost fabric fibers in some cases. The bright fragments observed after linear abrasion in (b) were aluminum oxide from the abrasive pad.

Mechanical durability is critical for functional textile coatings. To evaluate the adhesion and robustness of the Cu_2_O films, Cu_2_O-coated PP fabrics were subjected to five sequential tests simulating real-world stresses: ultrasonic washing, linear abrasion, adhesive tape peel, repeated twisting, and bending. After each test, the same sample was analyzed by SEM to evaluate coating integrity (Fig. 4a–e).

As shown in Fig. 4, the coatings remained largely intact across all tests. SEM images after washing showed no significant delamination or exposed fibers (Fig. 4a). In the abrasion test (Fig. 4b), a few bright debris particles were observed which were analyzed *via* energy-dispersive X-ray spectroscopy (EDX). A clear EDX signal from the debris particles was observed at ∼1.5 keV, confirming them as aluminum oxide from the abrasive pad, not detached Cu_2_O. No Al EDX signal was observed prior to the linear abrasion test. The tape peel test (Fig. 4c) removed only minimal material, with the Cu_2_O layer still visibly adhered. Even after twisting and repeated folding (Fig. 4d and e), only a small amount of damage was observed on the uppermost fabric fibers. This robustness is attributed to two key features of the coating: (1) its nanoscale thickness, which allows it to flex with the fabric, and (2) its conformal, shell-like coverage of fibers. By wrapping around the full circumference of each fiber, the film effectively anchors itself in place. This geometry reduces the likelihood of peeling unlike planar coatings which can lift easily from edges or scratches. These findings align with prior AP-SALD studies on ZnO-coated fabrics, which also showed strong mechanical resilience.^31^

#### 2.4.2. Aerosol penetration performance of Cu_2_O-coated fabrics

**Fig. 5:**
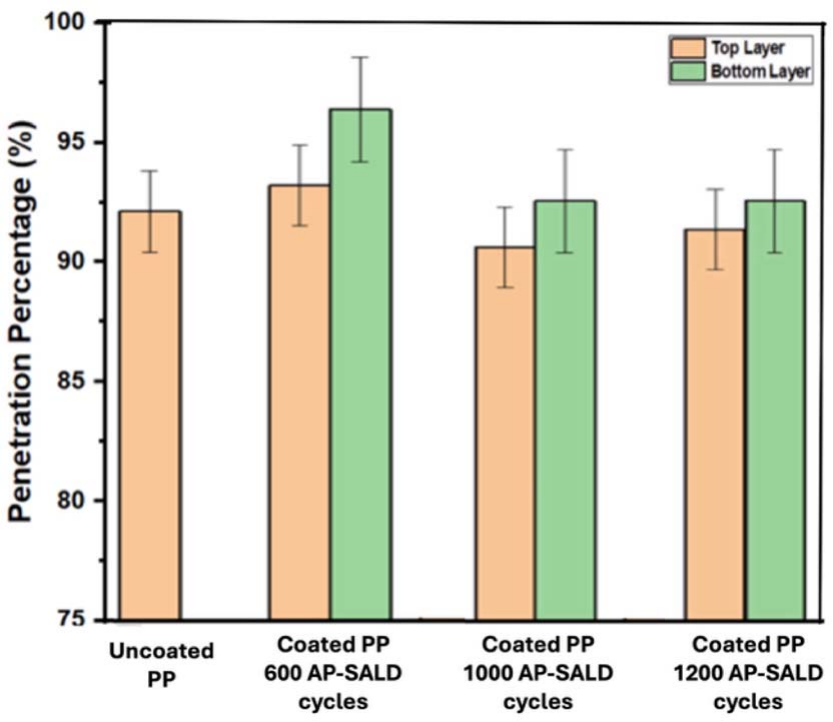
Penetration percentage of uncoated and coated PP fabric (50 g m^−2^). The test aerosol contains NaCl particles with count-median diameter of 0.075 ± 0.020 µm. An average of three measurements is presented with standard deviation indicated by error bars. All coated samples showed penetration values similar to the uncoated PP fabric.

For applications such as high-efficiency respirators, it is essential that functional coatings do not compromise particle filtration performance. To assess this, aerosol penetration tests were conducted following the NIOSH TEB-APR-STP-0059 protocol (additional details in the Experimental Methods Section). Fig. 5 summarizes NaCl-aerosol penetration through uncoated PP and Cu_2_O-coated fabrics prepared with 600, 1000, and 1200 AP-SALD cycles (approximately 30, 60, and 70 nm thick coatings, respectively, based on the GPC of 0.057 nm per cycle reported above for the top layer). The uncoated PP shows 92 ± 2% penetration, consistent with its role as an outer splash barrier rather than the primary filter in an N95 respirator. All coated samples showed penetration values between 90% and 96%, which falls within the experimental uncertainty (±2%) of the uncoated material. Slightly higher values were observed for some coated samples, particularly the bottom fabric layer, which is closer to the heated stage during deposition. This could be due to the heat affecting the morphology of the fabric. The negligible impact on filtration is consistent with the coating's nanoscale thickness and conformal morphology. The Cu_2_O film adds less than ∼0.1 µm to the surface of fibers approximately 20 µm in diameter, preserving the porosity and air pathways within the nonwoven structure. In addition, the coatings are strongly adhered, minimizing the risk of particle shedding that could contribute to filter loading or increased pressure drop. Airflow resistance measurements confirmed that the pressure drop across the fabric remained unchanged within ± 5% after coating. These findings are consistent with previous studies on ZnO-coated respirator fabrics, where coatings up to 100 nm thick also showed no measurable effect on aerosol penetration.^31^ Overall, the Cu_2_O functionalization does not degrade the filtration efficiency or breathability of the PP fabric.

#### 2.4.3. Biocompatibility of Cu_2_O-coated fabrics

**Fig. 6:**
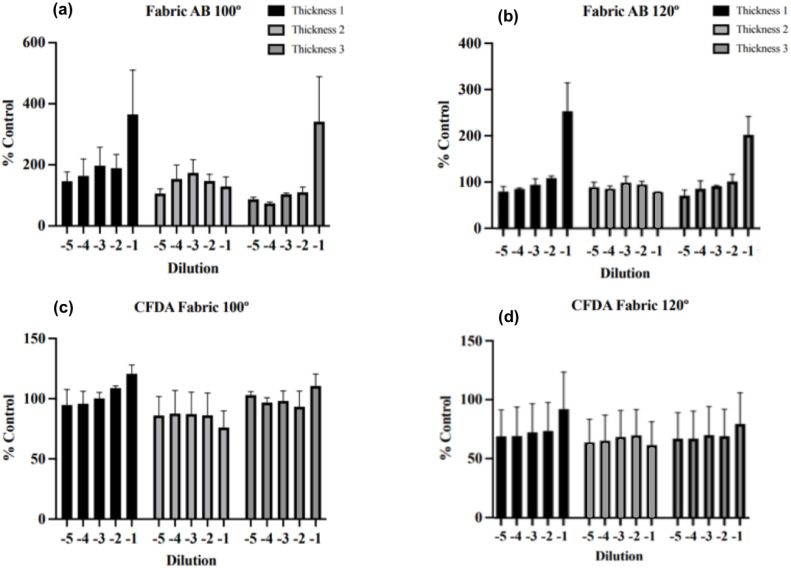
Cell viability following exposure to fabric extracts. HepG2 cells were treated with five increasing 1 : 10 dilutions of fabric extracts for 24 h, after which cell viability was measured using Alamar Blue (AB; a and b) and CFDA (c, d) with fabrics deposited at 100 °C (a and c) and 120 °C (b and d). Three thicknesses were tested. Results are presented as a % of untreated control cells, and represent the average of 3 independent experiments. Statistical comparisons were conducted using the Mann–Whitney *U* test, which indicated no significant changes.

Biocompatibility is crucial for practical application of healthcare and personal protective textiles. Cell viability was assessed with a dual-indicator assay in which Alamar Blue (AB) reports metabolic activity and 5-carboxyfluorescein diacetate (CFDA) reports membrane integrity.^33^ Hep G2 cells were exposed to fabric extracts across multiple dilutions (Fig. 6a–d). Fabrics coated at both 100 °C and 120 °C with varying Cu_2_O thicknesses were tested. “Thickness 1” (or T1) refers to the thinnest coating (∼30 nm), T2 to an intermediate thickness (∼60 nm), and T3 to the thickest Cu_2_O coating (∼70 nm).

Across all extract dilutions and both deposition temperatures there were no statistically significant changes in membrane integrity or cellular metabolism upon exposure with fabric extracts. CFDA fluorescence signals remained comparable to the uncoated control, indicating that the Cu_2_O coatings did not compromise cell membrane integrity. While not statistically significant, AB fluorescence did exhibit a notable increase at the highest extract concentration (least diluted). This elevated AB signal suggests stimulated cellular metabolism, most plausibly due to leaching of trace copper ions or other species into the media. This interpretation is supported by the stable CFDA results, which confirm that cell membranes remained intact and that the increase in metabolic activity was not accompanied by cytotoxic damage. With respect to film thickness, no significant differences were observed for the coatings: AB and CFDA responses for T1 (thin), T2 (intermediate), and T3 (thickest) samples clustered closely around the control values, suggesting minimal impact of coating thickness on biocompatibility at these test conditions. Collectively, these AP-SALD Cu_2_O coatings deposited at 100 °C and 120 °C provide excellent biocompatibility, meeting a key requirement for healthcare and personal protective applications.

## 3. Conclusion

In this work, AP-SALD was demonstrated as an effective, scalable method to deposit uniform, conformal Cu_2_O nanocoatings onto spun-bond polypropylene textiles, commonly used as outer layers in N95 respirators. By carefully controlling deposition parameters such as precursor flow, substrate temperature, and cycle number, continuous nanoscale Cu_2_O coatings were obtained without compromising the underlying polymeric substrates. Furthermore, *in situ* reflectance enabled real-time estimation of the nanoscale coating thickness.

The Cu_2_O coatings exhibited exceptional mechanical robustness, enduring various simulated real-world stresses including laundering, abrasion, bending, twisting, and adhesive peel testing. These tests confirmed minimal delamination, emphasizing strong film adhesion facilitated by the conformal, ultrathin nature of the ALD-derived coating. Additionally, critical textile functionalities like air filtration and breathability remained unaffected by the presence of the coatings. While further characterization, including particle-shedding tests, will be needed to ensure the coatings meet relevant standards, these preliminary results demonstrate the potential suitability of the coatings for integration into respiratory protective equipment.

Biocompatibility assessments using dual-indicator assays confirmed that coatings deposited at both 100 °C and 120 °C were non-cytotoxic. However, at the highest extract concentrations, elevated metabolic activity was detected suggesting potential copper-ion release. While detailed investigation into this phenomenon was beyond the scope of the current study, future work is recommended to optimize deposition parameters and study their impact on the microscopic coating properties to ensure both antimicrobial efficacy and safety in skin-contact applications.

Moving forward, longer-term studies on the stability of Cu_2_O coatings under real-use conditions will be essential to evaluate potential aging effects and future work can address cost-efficiency and precursor utilization through new reactor design. Overall, the outcomes of this research establish AP-SALD as a promising route for high-performance, multi-functional textile coatings. Notably, AP-SALD is an industrially scalable, roll-to-roll compatible technique that is currently being commercialized for high-throughput production in industries such as batteries, photovoltaics, and flexible consumer packaging.^22^ Hence, it is well-positioned for high-throughput, roll-to-roll manufacturing of functionalized fabrics. These Cu_2_O-functionalized fabrics combine mechanical durability, biocompatibility, and sustained filtration performance, making them highly attractive candidates for healthcare textiles, personal protective equipment and general consumer apparel.

## 4. Experimental methods

### 4.1 Deposition of Cu_2_O by AP-SALD

Cu_2_O was deposited on spun-bond polypropylene (50 g m^−2^) with a lab-scale AP-SALD tool equipped with a four-inlet, 21-slit reactor head. The copper precursor, Cu(i) bis-(hfac) trimethylvinylsilane (“Cupraselect”), was held at 50 °C in a stainless-steel bubbler to offset its low volatility, while water vapour served as the co-reactant. The Cupraselect was bubbled at 450 sccm with high-purity N_2_ and combined with a 50 sccm carrier flow. The water was bubbled at 200 sccm, again with high-purity N_2_ and combined with a 300 sccm carrier flow. Precursor streams were separated by 1000 sccm N_2_ curtains and the head-to-substrate gap was maintained at 100–150 µm. Rectangular PP pieces (8 × 7 cm^2^) were taped under slight tension to a heated borosilicate glass substrate. The substrate temperature was 100 °C or 120 °C (safely below PP's 160–170 °C melting point) and the stage oscillated under the reactor head at 1.5 cm s^−1^. Standard runs used 500 AP-SALD oscillations (1000 AP-SALD cycles), creating films about 60 nm thick, unless stated otherwise.

### 4.2 Characterization

SEM images and EDX data were collected using a Zeiss Ultra Plus field emission SEM at an accelerating voltage of 5 kV. Crystalline phases were analyzed by XRD on a Bruker D2 Phaser diffractometer with Cu Kα radiation (*λ* = 1.5406 Å), operated in grazing-incidence mode. XPS was performed using a Thermo VG Scientific ESCALab 250 Imaging XPS Microprobe with Al Kα excitation, and spectra were analyzed with CasaXPS software to determine copper oxidation states and surface composition. Optical absorption spectra of Cu_2_O films deposited on glass were recorded using a Horiba QuantaMaster 8000 UV-vis spectrophotometer to estimate the optical bandgap *via* Tauc analysis. Additionally, *in situ* reflectance measurements during deposition were carried out using an Ocean Optics QR600 reflectance probe, DH-2000 UV-vis light source, and HDX-UV-VIS spectrometer, spanning 250–800 nm.

### 4.3 Mechanical and adhesion tests

To assess coating durability, coated fabrics underwent five mechanical stress tests. Washing was performed by stirring samples in 200 mL of water with 1.0 g of “Gain” detergent at 40 °C and 450 rpm for 30 minutes, followed by two rinses in distilled water (15 minutes each) and drying at 120 °C for 1 hour. For abrasion, the fabric was placed face-down on 180-grit sandpaper with a 40 g weight on top and moved back and forth over 10 cm at a ∼30° angle, repeated 15 times. In the twisting test, one end of the fabric was taped to a benchtop while the other end was twisted 360° back and forth for 50 cycles. Tape adhesion was tested by applying Scotch tape to the fabric under a 150 g weight and peeling it off, repeated twice. For bending, a labeled region of the fabric was wrapped around a 4.5 mm radius rod and bent 300 times. Post-test analysis was conducted *via* SEM to identify any coating damage or delamination.

### 4.4 Aerosol penetration testing

An Automated Filter Tester (CERTITEST, Model 8130A, TSI, Inc.) was used to quantify particle penetration. Single layers of coated or uncoated spun-bond PP were cut into square sections and clamped in a custom acrylic adapter with a square 5 × 5 cm (25 cm^2^) testing area (necessary because the instrument's standard 14 cm holder exceeded the lab-scale coating area). A charge-neutralized NaCl aerosol with a count-median diameter of 0.075 ± 0.020 µm was generated. The National Institute for Occupational Safety and Health (NIOSH) protocol was followed. To maintain the NIOSH face velocity of ≈10.77 cm s^−1^, the volumetric flow rate was adjusted to 16.00 L min^−1^. Upstream and downstream aerosol concentrations were recorded simultaneously by dual light-scattering photometers, and penetration (%) was calculated from their ratio. Three measurements were performed for each fabric; both the top and bottom layers produced by the two-layer AP-SALD process were tested separately. The penetration of the uncoated outer PP layer provided a baseline. The average penetration for each fabric is plotted in Fig. 5, with the standard deviation indicated by error bars.

### 4.5 Cell viability assays

To assess the biocompatibility of the Cu_2_O-coated fabrics, cell viability tests were conducted using a dual fluorescent dye approach with Alamar Blue and 5-carboxyfluorescein diacetate. Cell growth media (DMEM, 10% fetal bovine serum purchased from VWR (Hyclone) and 1% penicillin-streptomycin) were first incubated with the coated fabric samples for 24 h to generate extracts, which were then serially diluted 1 : 10–1 : 100 000, and applied to Hep G2 cells obtained from the American Type Culture Collection (ATCC, HB-8065; 3 × 10^4^ cells per well; 96-well plates). After a 24 h exposure, cells were incubated for 1 hour at 37 °C with 10% AB and 4 µM CFDA, and fluorescence was measured using a Synergy HT plate reader (BioTek) at excitation/emission wavelengths of 530/590 nm (AB) and 485/528 nm (CFDA). Viability was calculated as a percentage of control (cells with extract from uncoated PP). Two sets of coatings were tested (deposited at 100 °C and 120 °C), each at three thickness levels (approximately 45, 60, and 100 nm thick coating). Cell viability assays were conducted in three independent experiments.

## Author contributions

G. Gurbandurdyyev: formal analysis, investigation, methodology, writing – original draft. S. Khalid: formal analysis, writing – original draft. S. Lum: formal analysis, investigation, methodology. F. Ye: investigation. A. Cheon: formal analysis, investigation, methodology, writing – review & editing. K. C. Tam: funding acquisition, methodology, supervision, writing – review & editing. S. DeWitte-Orr: funding acquisition, methodology, supervision, writing – review & editing. K. P. Musselman: conceptualization, funding acquisition, methodology, supervision, writing – review & editing.

## Conflicts of interest

There are no conflicts of interest to declare.

## Supplementary Material

NA-OLF-D6NA00121A-s001

## Data Availability

The supporting data for this study have been included in the article or as part of the supplementary information (SI). Supplementary information: pictures of coating one, two, or three layers of fabric on glass, schematics of subsurface infiltration, cross-section SEM images of Cu_2_O-coated PP fabric, and characterization of Cu_2_O coatings deposited at different temperatures (XRD, UV-vis spectroscopy, XPS). See DOI: https://doi.org/10.1039/d6na00121a.

## References

[cit1] Hyde G. K., Scarel G., Spagnola J. C., Peng Q., Lee K., Gong B., Roberts K. G., Roth K. M., Hanson C. A., Devine C. K., Stewart S. M., Hojo D., Na J. S., Jur J. S., Parsons G. N. (2010). Langmuir.

[cit2] Jur J. S., Sweet III W. J., Oldham C. J., Parsons G. N. (2011). Adv. Funct. Mater..

[cit3] Hussien A. M., Gawish S. M., Ramadan A. M., Mosleh S., Sayed G. H. (2024). Egypt. J. Chem..

[cit4] Zoolfakar A. S., Rani R. A., Morfa A. J., O'Mullane A. P., Kalantar-Zadeh K. (2014). J. Mater. Chem. C.

[cit5] Tortella G. R., Pieretti J. C., Rubilar O., Fernández-Baldo M. A., Benavides-Mendoza A., Diez M. C., Seabra A. B. (2022). Crit. Rev. Biotechnol..

[cit6] Imani S. M., Ladouceur L., Marshall T., Maclachlan R., Soleymani L., Didar T. F. (2020). ACS Nano.

[cit7] Gonçalves R. A., Ku J. W. K., Zhang H., Salim T., Oo G., Zinn A. A., Boothroyd C., Tang R. M. Y., Gan C. L., Gan Y.-H., Lam Y. M. (2022). ACS Appl. Nano Mater..

[cit8] Ghosh J., Rupanty N. S., Noor T., Asif T. R., Islam T., Reukov V. (2025). RSC Adv..

[cit9] Ali A., Petrů M., Azeem M., Noman T., Masin I., Amor N., Militky J., Tomkova B. (2023). J. Ind. Text..

[cit10] Sfameni S., Hadhri M., Rando G., Drommi D., Rosace G., Trovato V., Plutino M. R. (2023). Inorganics.

[cit11] Tan X.-Q., Liu J.-Y., Niu J.-R., Liu J.-Y., Tian J.-Y. (2018). Materials.

[cit12] Qiao S., Shi Z., Tong A., Luo Y., Zhang Y., Wang M., Huang Z., Xu W., Chen F. (2025). Adv. Colloid Interface Sci..

[cit13] Karttunen A. J., Sarnes L., Townsend R., Mikkonen J., Karppinen M. (2017). Adv. Electron. Mater..

[cit14] Shaban M., Zayed M., Hamdy H. (2017). RSC Adv..

[cit15] Nodoushan R. M., Shekarriz S., Shariatinia Z., Montazer M., Heydari A. (2023). Surf. Coat. Technol..

[cit16] Puurunen R. L. (2005). J. Appl. Phys..

[cit17] Huang M. L., Cai Z., Wu Y. Z., Lu S. G., Luo B. S., Li Y. H. (2020). Vacuum.

[cit18] Chen H.-T., Huang M.-C., Chiang Y.-Y., Chang Y., Wu C.-C. (2025). Mater. Adv..

[cit19] Pollard Z. A., Karod M., Goldfarb J. L. (2021). Sci. Rep..

[cit20] Poodt P., Lankhorst A., Roozeboom F., Spee K., Maas D., Vermeer A. (2012). J. Vac. Sci. Technol., A.

[cit21] Musselman K. P., Uzoma C. F., Miller M. S. (2016). Chem. Mater..

[cit22] Hoye R. L. Z., Muñoz-Rojas D., Sun Z., Okcu H., Asgarimoghaddam H., MacManus-Driscoll J. L., Musselman K. P. (2025). PRX Energy.

[cit23] Delumeau L. V., Asgarimoghaddam H., Alkie T. N., Jones A. J. B., Lum S., Mistry K., Aucoin M. G., DeWitte-Orr S. J., Musselman K. P. (2021). APL Mater..

[cit24] Musselman K. P., Muñoz-Rojas D., Hoye R. L. Z., Sun H., Sahonta S.-L., Croft E., Bohm M. L., Ducati C., MacManus-Driscoll J. L. (2017). Nanoscale Horiz..

[cit25] Wilson C. A., Grubbs R. K., George S. M. (2005). Chem. Mater..

[cit26] Jur J. S., Spagnola J. C., Lee K., Gong B., Peng Q., Parsons G. N. (2010). Langmuir.

[cit27] Jung S., Yang J.-Y., Byeon E.-Y., Kim D.-G., Lee D. G., Ryoo S., Lee S., Shin C. W., Jang H. W., Kim H. J., Lee S. (2021). Polymers.

[cit28] Guillén C., Herrero J. (2018). J. Alloys Compd..

[cit29] Muñoz-Rojas D., Jordan M., Yeoh C., Marin A., Kursumovic A., Dunlop L. A., Iza D., Chen A., Wang H., MacManus-Driscoll J. L. (2012). AIP Adv..

[cit30] Piegari A., Masetti E. (1985). Thin Solid Films.

[cit31] Gurbandurdyyev G., Mistry K., Delumeau L.-V., Loke J. Y., Teoh C. H., Cheon J., Tam K. C., Musselman K. P. (2023). ChemNanoMat.

[cit32] Breiland W. G., Killeen K. P. (1995). J. Appl. Phys..

[cit33] Schreer A., Tinson C., Sherry J. P., Schirmer K. (2005). Anal. Biochem..

