# Supplementary material for: Robust, conformal Cu2O coatings on polypropylene fabrics via atmospheric-pressure spatial atomic layer deposition
Source: Nanoscale Adv. 2026 May 1;8(11):3386–95. doi: 10.1039/d6na00121a (PMC13159011; doi:10.1039/d6na00121a)
Supplement: NA-008-D6NA00121A-s001 [file NA-008-D6NA00121A-s001.pdf]

Supplementary Information (SI) for Nanoscale Advances.
This journal is © The Royal Society of Chemistry 2026

# Supporting Information

**Robust, Conformal $Cu_2O$ Coatings on Polypropylene Fabrics via Atmospheric-Pressure Spatial Atomic Layer Deposition**

*Guvanch Gurbandurdyyev, Sarah Khalid, Samantha Lum, Fan Ye, Autumn Cheon, Kam Chiu Tam, Stephanie DeWitte-Orr, and Kevin P. Musselman**

*Corresponding author: kevin.musselman@uwaterloo.ca

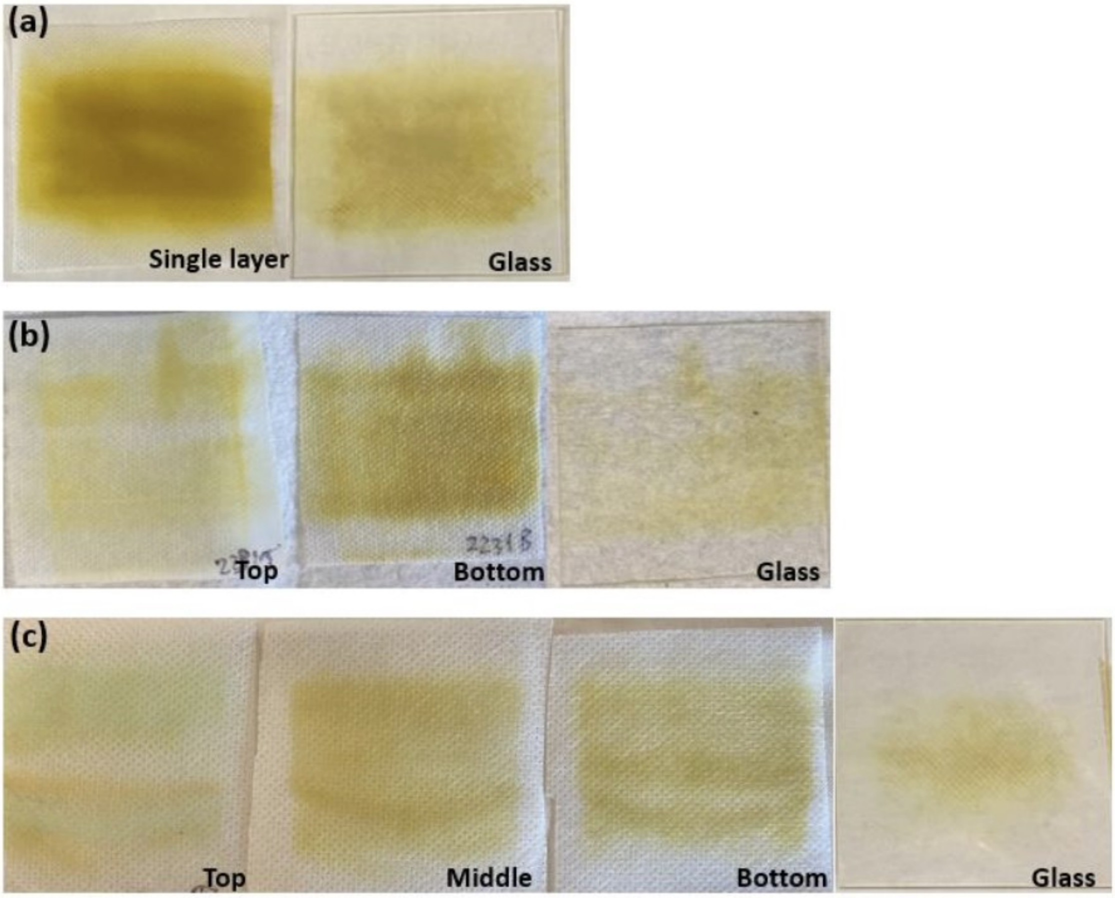


***Figure S1.*** *Coating of (a) one layer, (b) two layers, and (c) three layers of PP fabric on glass (7 cm x 7cm) with* $Cu_2O$.

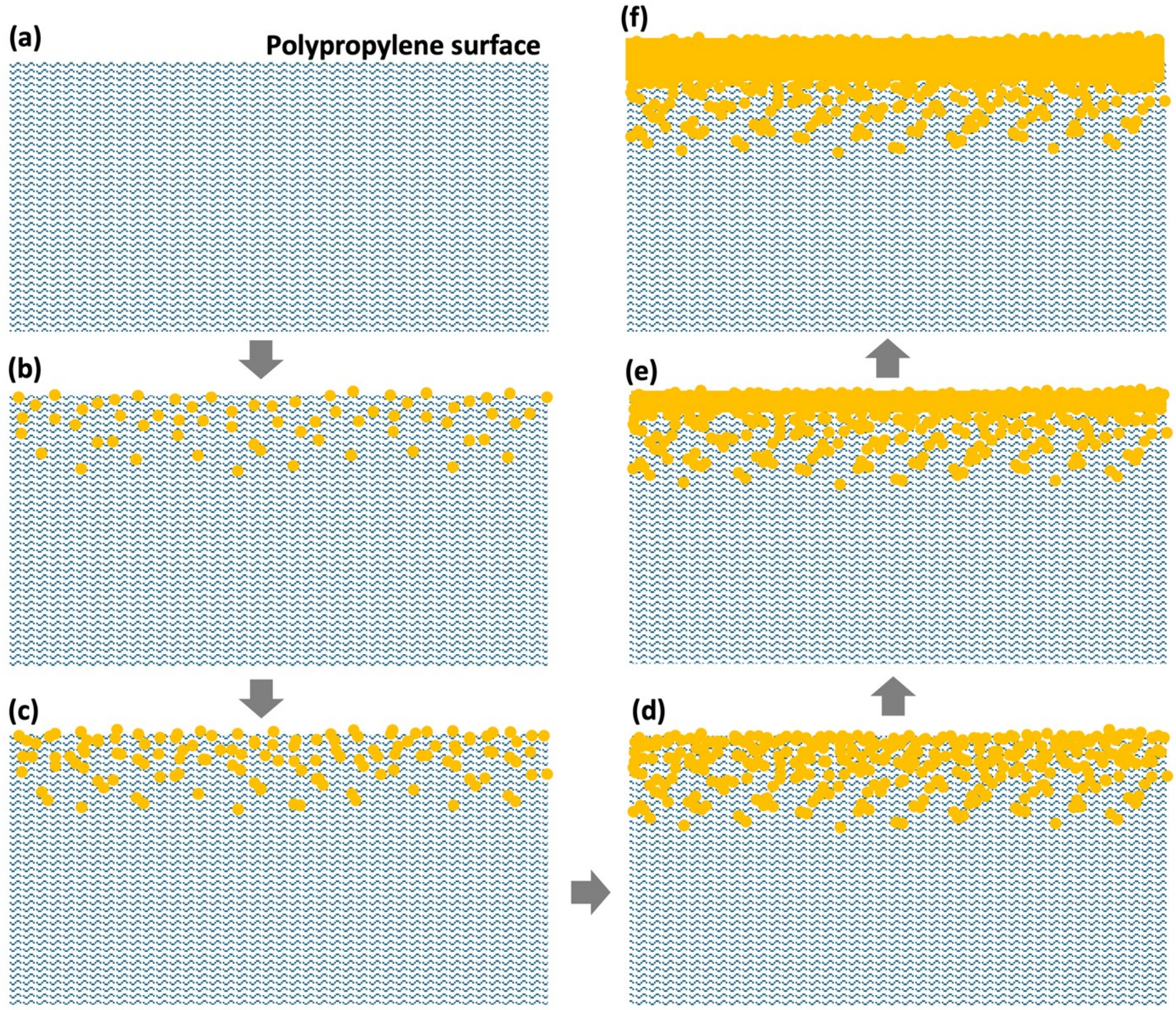


***Figure S2.*** *Illustration of subsurface infiltration process. (a) Surface of polypropylene fiber. (b) The chemical precursors, Cupraselect and water, get trapped in the near-surface region, forming $Cu_2O$ nucleation clusters. (c-d) The $Cu_2O$ clusters coalesce into larger clusters that fill the space between the polymer chains. (e-f) Cluster coalescence results in the formation of a continuous $Cu_2O$ film that grows on the polypropylene surface.*

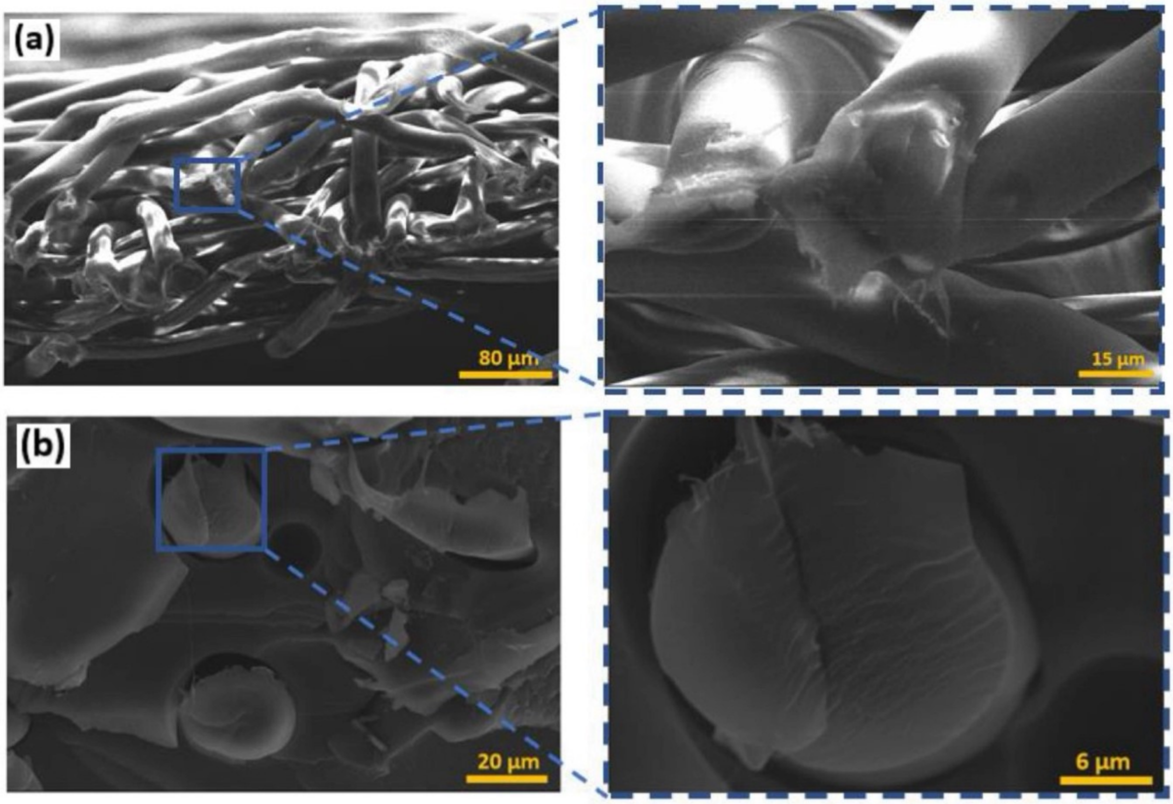


***Figure S3.*** *Cross-section SEM image of $Cu_2O$-coated PP fabric (600 AP-SALD oscillations) (a) after immersed in liquid nitrogen, and (b) after encapsulated with epoxy.*

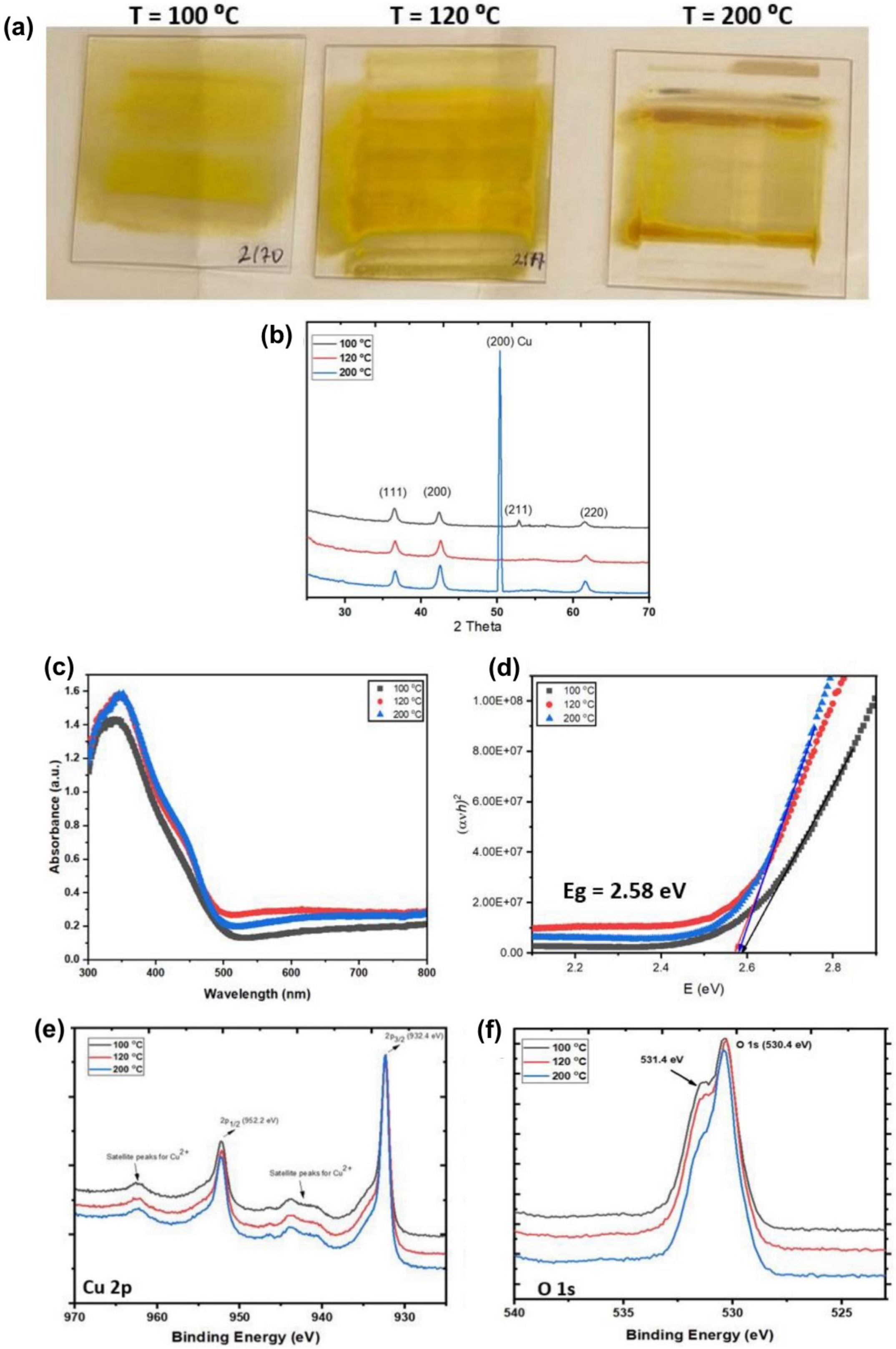


***Figure S4.*** *(a) The $Cu_2O$ thin-film coatings deposited at 100 °C, 120 °C, and 200 °C, (b) their XRD patterns, (c) UV-Vis absorbance spectra, and (d) corresponding Tauc plots with calculated optical band gap energy value ($E_g$). XPS measurement of $Cu_2O$ samples showing (e) Cu 2p and (f) O 1s spectra.*
